# A Textile Waste Fiber-Reinforced Cement Composite: Comparison between Short Random Fiber and Textile Reinforcement

**DOI:** 10.3390/ma14133742

**Published:** 2021-07-04

**Authors:** Payam Sadrolodabaee, Josep Claramunt, Mònica Ardanuy, Albert de la Fuente

**Affiliations:** 1 Department of Civil and Environmental Engineering, Polytechnic University of Catalonia, 08034 Barcelona, Spain; albert.de.la.fuente@upc.edu; 2 Department of Agricultural Engineering, Polytechnic University of Catalonia, 08034 Barcelona, Spain; josep.claramunt@upc.edu; 3 Department of Material Science and Engineering, Polytechnic University of Catalonia, 08222 Barcelona, Spain; monica.ardanuy@upc.edu

**Keywords:** cementitious materials, fiber-reinforced composites, mechanical properties, recycled fibers, sustainability, textile waste

## Abstract

Currently, millions of tons of textile waste from the garment and textile industries are generated worldwide each year. As a promising option in terms of sustainability, textile waste fibers could be used as internal reinforcement of cement-based composites by enhancing ductility and decreasing crack propagation. To this end, two extensive experimental programs were carried out, involving the use of either fractions of short random fibers at 6–10% by weight or nonwoven fabrics in 3–7 laminate layers in the textile waste-reinforcement of cement, and the mechanical and durability properties of the resulting composites were characterized. Flexural resistance in pre- and post-crack, toughness, and stiffness of the resulting composites were assessed in addition to unrestrained drying shrinkage testing. The results obtained from those programs were analyzed and compared to identify the optimal composite and potential applications. Based on the results of experimental analysis, the feasibility of using this textile waste composite as a potential construction material in nonstructural concrete structures such as facade cladding, raised floors, and pavements was confirmed. The optimal composite was proven to be the one reinforced with six layers of nonwoven fabric, with a flexural strength of 15.5 MPa and a toughness of 9.7 kJ/m^2^.

## 1. Introduction

The building sector is one of the major consumers of natural resources and one of the biggest waste producers worldwide. Data indicate that the construction and building sector consumes almost 40% of all raw materials extracted worldwide and is responsible for around 40% of all global greenhouse gas emissions in addition to the generation of around 35% of all global waste [1,2]. Therefore, gradual replacement of the traditional linear economy model with a circular material flow approach focused on reusing and recycling is necessary to ensure a sustainable future [3].

The building sector is increasingly interested in innovative sustainable solutions, i.e., materials obtained from recycling and reusing processes so that CO_2_ emissions and energy intake can be reduced [4]. In this regard, fiber- and textile-reinforced mortars (FRM and TRM, hereinafter) have generated great interest among both the scientific and construction sectors. These composite materials may be composed of various materials for reinforcement—short fibers [5], long fibers [6], and textile including woven [7] or nonwoven fabrics [8]—within a cementitious matrix, which can be in the form of cement paste, lime binder, mortar, or concrete. The primary role of reinforcement is to bridge cracks as well as to enhance the toughness, energy absorption capacity, and post-cracking behavior of cementitious matrices [5,9].

World fiber production, including in steel, glass, and polymers, has been steadily increasing in the past few decades and has garnered increasing interest with the use of sustainable fibers produced from renewable, biodegradable, waste, recycled, available, and low-cost resources becoming a focal point. In this sense, vegetable and cellulosic fibers have already been used as reinforcement in cementitious materials for low- to medium-performance structural applications [5,10,11,12,13]. Textile waste fiber could be another sustainable alternative for reinforcement in cementitious composites.

The global production of textiles amounts to over 110 million tons annually, which makes textile production one of the biggest industries affecting global environmental pollution through greenhouse gas emissions, depletion of natural resources, and the generation of huge amounts of waste [3]. Textile leftovers can be categorized as pre- or post-consumer waste, where the former includes all fiber, yarn, and fabric waste produced during garment manufacturing while the latter refers to worn-out clothing discarded by users [14]. In Europe and America, more than 10 million tons of discarded textile products are disposed of into landfills [15], and the estimation for China is double this amount, which implies serious environmental and economic issues. Nevertheless, the rate of textile waste recycling is rather low at less than 20%; 95% of this waste material has recyclability potential [16]. The use of textile waste (TW, hereinafter) in cementitious composites as an alternative material for reinforcement is therefore a promising option for reusing this waste.

TW fibers can be made of natural or human-made fibers including cotton, silk, flax, polypropylene, nylon, and polyester, all of which have a lower elastic modulus than the matrix. According to several studies [17,18,19,20,21,22], TW fibers from polyester and nonwoven fabrics can be used as thermal- and sound-insulating elements. Moreover, lightweight bricks, cement blocks, and concrete partitions containing TW fiber, namely cotton, are already being produced [23,24,25,26]. In addition, textile effluent sludge is being reused in non-load-bearing concrete blocks [27].

Regarding the mechanical properties of TW fiber-reinforced concrete, some studies have investigated concrete reinforced with nylon or polypropylene fibers recycled from carpet [28]. In the literature, the engineering of concrete has, in most cases, enhanced properties such as tensile and flexural strength whilst others such as compressive strength, workability, and elastic modulus have declined [29,30,31,32,33]. The inclusion of recycled textile fibers was observed to influence the mechanical performance of concrete through a bridging action against crack propagation and redistribution of the porous matrix structure toward a more uniform structure [28,34]. Nonetheless, the use of a high dosage of waste fibers leads to an agglomeration effect which, in turn, causes the formation of voids and entrapped air, thereby diminishing the concrete’s properties [35].

Furthermore, the effect of textile waste cuttings from garments on the mechanical properties of polymer concrete was investigated in [36]. The results showed that the addition of TW fibers with lengths between 2 and 6 cm eliminates the brittleness of unreinforced polymer concrete, thereby leading to smoother failure, although no considerable enhancement in flexural and compressive resistance was observed.

It is widely believed that the incorporation of fibers can improve the shrinkage behavior of cementitious materials. Shrinkage cracks of restrained cementitious materials can be a problem in terms of aesthetics and durability since water, chlorides, and other harmful minerals could enter those cracks, causing early deterioration and damage. Thus, controlling shrinkage cracks is of paramount importance for improving service life and minimizing repair costs [37]. The majority of available research on the addition of vegetable or synthetic fibers suggests that they have a favorable effect in minimizing the plastic and autogenous shrinkage of cement composites [37,38,39,40].

However, the drying shrinkage of a fiber-reinforced cement-based composite has scarcely been reported, and the results are inconclusive. Toledo et al. [40] and Silva et al. [41] investigated the drying shrinkage of a matrix consisting of fine aggregate and supplementary cementitious materials reinforced with short and long sisal fibers. The conclusion was that shrinkage increased with respect to the reference sample as the addition of vegetable fibers increased matrix porosity, thereby contributing to higher drying shrinkage of the composite. In other studies [42,43,44], it was reported that the addition of low contents of pulp and cellulose fiber could reduce drying shrinkage and thereby mitigate related cracking in the concrete. Moreover, Wang et al. [45] and Mohammadhosseini et al. [46] concluded that the inclusion of recycled polypropylene carpet fibers in concrete reduced the drying shrinkage of the control by up to 30% due to the interruption of moisture transfer from the internal microstructure of the cementitious matrix to the external environment.

According to our literature review, the mechanical properties of cement-based composites reinforced with short TW from garment resources of cotton and polyester have not been comprehensively investigated. Furthermore, research on nonwoven fabric in cementitious mortars as reinforcement remains scarce [8,12,47], whilst the majority of studies cover other different woven textile forms including glass, carbon, and vegetal fabrics [48,49,50,51,52,53,54,55,56,57] as well as long fibers including sisal strands [58,59,60]. Nonetheless, all studies have concluded that TRMs with thin and lightweight composites have enhanced flexural, tensile, and strain–hardening behaviors.

In view of the abovementioned, two experimental programs were carried out to evaluate the properties of engineered TW-reinforced cement composites. One involved the use of a fraction of short randomly dispersed TW fiber in contents ranging from 6 to 10% by weight in cement [61], while the other was focused on textile lamination of nonwoven fabrics, ranging from 3 to 7 layers of this fiber [62]. The goal of this scientific contribution is to analyze and compare the results obtained from these experimental programs—including flexural resistance in pre- and post-crack, toughness, stiffness, durability, and shrinkage—to identify the most suitable composite for potential application in building construction.

## 2. Materials and Methods

### 2.1. Materials

Portland cement type I 52.5R, with physical and chemical properties reported in [62] and supplied by Cementos Molins Industrial, S.A. (Barcelona, Spain), was used to produce the pastes in all samples.

TW short fibers from clothing waste were provided by Triturats La Canya S.A. (Girona, Spain) and consisted of almost 31% polyester and 69% cotton, two prevailing types of fiber in the global market. As reported in [61], the water retention value and moisture content of the fiber were 85 and 7%, respectively. Furthermore, the majority of these short fibers had a diameter ranging from 3.6 to 32.1 µm, while the rest was a mix of yarns and fabrics.

**Figure 1:**
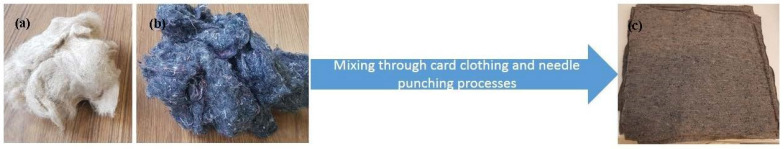
(**a**) Flax fiber; (**b**) textile waste fiber; (**c**) final nonwoven fabric.

As the production of nonwoven fabric from 100% TW fiber failed due to fibers being too short, longer flax fibers (F, hereinafter) with an average length of 60 mm, provided by Instytut Wlokien Naturalnych (Poznań, Poland), were mixed with TW fibers. Each TW nonwoven fabric (see Figure 1), with dimensions of 0.75 mm × 300 mm × 300 mm and an areal weight of 155 g/m^2^, was composed of 65% TW and 35% F fibers. Thus, the TW nonwoven fabric consisted of almost 80% vegetable fibers (35% Flax and 45% cotton) and 20% synthetic fiber. The production of nonwoven fabric, including card clothing and needle-punching, has been described in depth in [62]. The maximum tensile rupture load (per weight) of the TW nonwoven mats was reported as 2.0 N/g.

### 2.2. Sample Preparation

**Figure 2:**
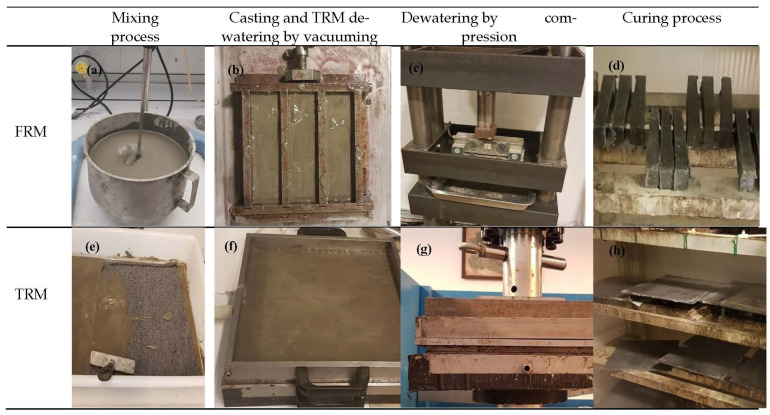
Preparation of the samples: (**a**) mixing process; (**b**) FRM casting; (**c**) dewatering of the FRM; (**d**) curing condition of the FRM; (**e**) impregnation of the nonwoven fabric with the paste; (**f**) TRM casting and vacuuming; (**g**) dewatering of the TRM; and (**h**) curing condition of the TRM plates. (FRM and TRM: Fiber- and Textile-Reinforced Mortar)

The FRM, a mortar reinforced with short TW fiber, was prepared in a laboratory mixer pan (Velp scientifica, model LS, Usmate, Italy) and cast into a 20 mm × 40 mm × 160 mm mold in which 5 MPa pressure was applied for 24 h to eliminate excess water. The TRM, a mortar reinforced with nonwoven textile, was prepared as plates of nonwoven layers impregnated with the cement paste placed cross-oriented in a mold with internal dimensions of 10 mm × 300 mm × 300 mm that underwent a homogeneous pressure of 3.3 MPa. The dewatering process for TRM plates included vacuuming (Vacuum pump, Matest, Treviolo, Italy) as well as compressing the mold for 24 h. All samples of FRM and TRM were cured for 28 days at ambient temperature (20 °C) in a humidity chamber (approximately 95% of relative humidity). Figure 2 depicts the process of preparation and casting of both composites.

The designation of the specimens (Table 1) was based on the fiber dosage of the cement weight for FRM, (6, 8, and 10%) and the number of reinforcement layers for TRM (3–7). The samples used for durability tests were those whose code ends in D. The final water/cement ratios (the final amount of water was calculated after weighing the amount of water eliminated by the dewatering treatment) together with the dosage of the materials—for FRM, as related to 1000 cm^3^ of mortar—are also reported in Table 1. The initial water/cement ratio for preparing the paste was established as 1.0 and 0.5 for TRM and FRM, respectively. Six specimens were cast for each code of FRM, while for each plate of TRM, six specimens were machined.

### 2.3. Flexural Tensile Strength Test and Toughness

**Figure 3:**
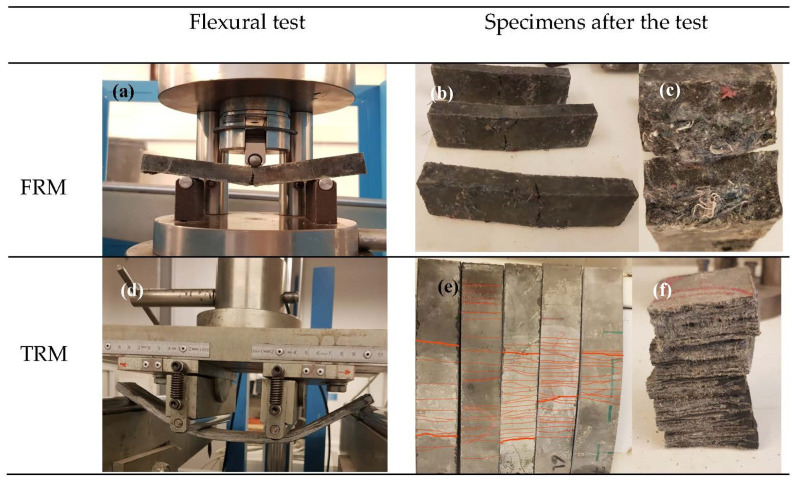
Flexural tests setup: (**a**) FRM three-point bending test; (**b**) cracks of FRM specimens; (**c**) cross-section of FRM specimens; (**d**) TRM four-point bending test; (**e**) cracks of TRM specimens; and (**f**) cross-section of TRM specimens.

Three-point (3P) flexural tests based on EN 12467:2012 [63] (Figure 3a–c) using an INCOTECNIC press machine (INCOTENIC UTM, Castelldefels, Spain) equipped with a load cell of 3 kN capacity and a loading rate of 4 mm/min on 100 mm span-length FRM specimens were carried out to identify the extent of the fiber’s contribution to bridging cracks. The mechanical properties of the TRM composites were determined under a four-point (4P) bending test configuration (Figure 3d–f) following RILEM TFR1 and TFR 4 [64]. An INCOTECNICpress equipped with a maximum load cell of 3 kN with a crosshead speed of 20 mm/min with a major span (L) of 270 mm was used.

The maximum flexural tensile strengths (also named modulus of rupture, MOR) of the FRM and TRM composites were determined using Equations (1) and (2), respectively, where Pmax is the maximum load recorded, L is the span length, and b and h are the cross-sectional width and thickness, respectively.

(1) $${\mathrm{MOR}}_{3P}=\frac{3P_{\max}\times L}{2b\times h^{2}}$$

(2) $${\mathrm{MOR}}_{4P}=\frac{P_{\max}\times L}{b\times h^{2}}$$

The toughness index (I_G_) was established as the reference parameter to characterize the type of failure (ductile or brittle) and the post-cracking deformation capacity. This parameter, based on the previously mentioned RILEM documents, TFR1 and TFR 4, is defined as the area beneath the force-displacement curve derived from the flexural test and values range from 0 to 0.4 MOR or the deformation value corresponding to 10% of the span, depending on which occurs first. For FRM samples, the limitation of 40% MOR dominated, while for the TRM composites, the limitation of 10% of the displacement value (27 mm) occurred first. This method has been previously used in other studies [5,12,47,62].

The flexural stiffness or modulus of elasticity in the pre-cracked zone (K) was also measured from the force-displacement relationships within the elastic regime using Equations (3) and (4) for FRM and TRM, respectively. In these equations, ∆P and ∆f are the variations in forces and deflections of two points in the linear-elastic state, and the rest of the parameters have already been defined.

(3) $$K_{3P}=\frac{\Delta P\times L^{3}}{4\Delta f\times bh^{3}}$$

(4) $$K_{4P}=\frac{23\Delta P\times L^{3}}{108\Delta f\times {\mathrm{bh}}^{3}}$$

### 2.4. Durability Test and Microscope Analyses

Among the different durability tests, resistance against dry-wet cycles is considered a challenge for cement-based composites reinforced with vegetable fibers [65,66]. As the short TW fiber and the fabric form consisted of vegetable fiber, cotton, and flax, the durability of the composite subjected to accelerated aging was investigated. To this end, those composites displaying better unaged mechanical properties were subjected to 25 dry-wet cycles after 28 days of curing. Each dry-wet cycle consisted of drying for 6 h at 60 °C and 60% of RH (Relative Humidity) followed by 18 h of immersion in water at 20 °C according to EN 12467:2012. In fact, repeated wetting-drying cycles simulated natural weathering conditions and could allow for a rough estimate of the durability of the composites.

To analyze the fractured surface microstructure and the effects of the dry-wet cycles, observations were made from scanning electron microscope images (Jeol JSM 5610 SEM, Jeol Ltd., Tokyo, Japan).

### 2.5. Drying Shrinkage Test

**Figure 4:**
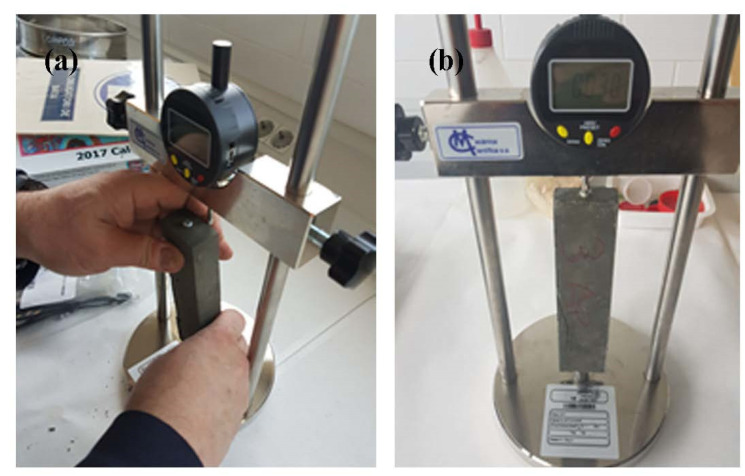
Shrinkage test: (**a**) shrinkage test setup; (**b**) monitoring the change in length.

In this study, the free drying shrinkages of the reference sample—cement paste only, without any fiber—and the FRM and TRM samples were measured using a digital micrometer (Matest, model E078KIT, Treviolo, Italy) to monitor the change in length at room temperature (see Figure 4). Shrinkage measurement started after 28 days of curing until reaching the maximum value. Microstrain shrinkage was computed using Equation (5), where ΔL_sh_ is the contraction of the length and L_0_ is the initial length of the specimen.

(5) $$\varepsilon_{\mathrm{sh}}=\frac{\Delta L_{\mathrm{sh}}}{L_{0}}\times 10^{6}$$

## 3. Results and Discussion

### 3.1. Flexural Test on Unaged Composites

**Figure 5:**
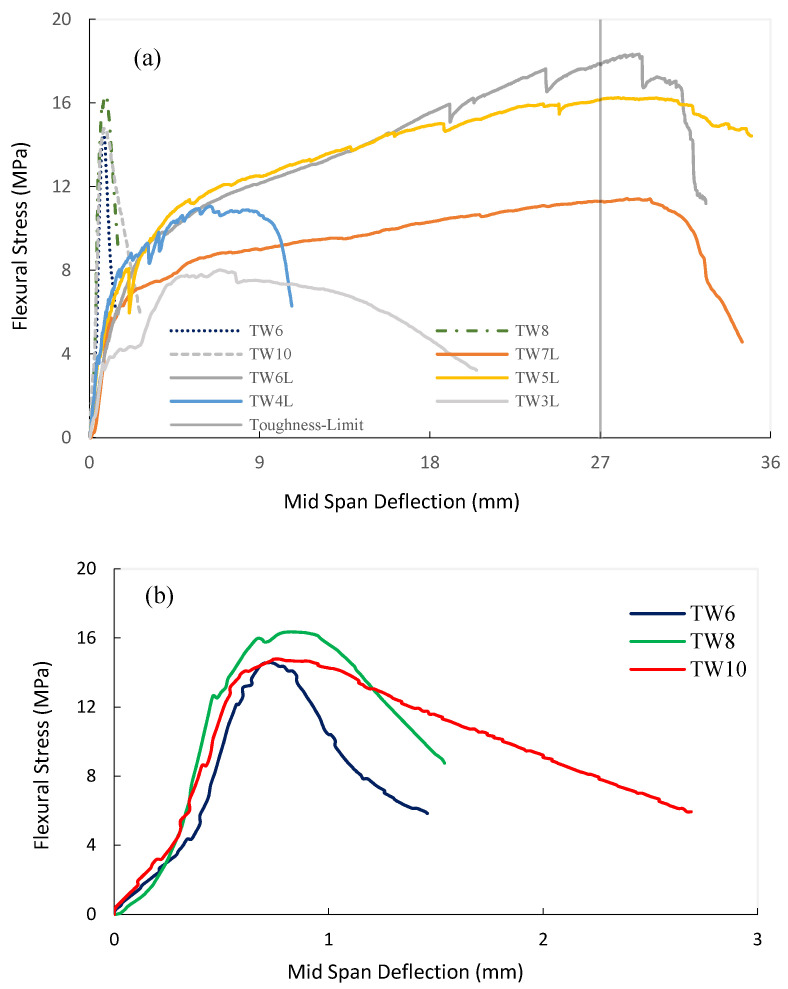
Representative flexural stress: deflection relationships obtained at 28 days for (**a**) all samples and (**b**) FRM samples.

On the one hand, the results depicted in Figure 5a suggest that TRM specimens show significantly greater post-failure energy absorption capacities under flexure than FRM specimens due to the multiple cracking patterns generated in the former. The bending response of TRM specimens could be divided into four distinct branches: (1) A linearly ascending branch, in which the external load was mainly borne by the cement matrix until a visible crack in the cement matrix was formed when the LOP (limit of proportionality) for strength was reached. (2) Crack propagation occurred along with multiple cracking formations. In this transition zone, the matrix contributed to the composite’s strength at non-cracked zones (tension stiffening) whilst the reinforcement’s contribution dominated the cracked zones. In the zones between cracks, the stress transfer mechanism was guaranteed by the reinforcement-matrix adhesion. (3) In another ascending branch (post-cracking), with the lowest slope due to degradation of the composites’ stiffness, the fabric reinforcement bridged the cracks and bore the loads. No further new cracks occurred in this zone, and cracks grew only in width. (4) Finally, failure occurred due to rupture or debonding of the fibers followed by further widening of the cracks and, eventually, due to the concentration of damage in a single crack.

On the other hand, FRM specimens (Figure 5a,b) showed rather brittle responses once cracking began. In this regard, the bending response of FRM specimens could be divided into three distinct branches: (1) an elastic range for the pre-cracking zone, as observed in TRM; (2) a post-cracking regime with a reduced number of cracks (1–2) leading to a significantly smaller deformation capacity with respect to TRM; and (3) pre- and post-failure branches comprising less than 2 mm of deflection (from the cracking onset) and, hence, limiting both the ductility and energy absorption capacity and being insufficient for the majority of structural applications for building.

According to the results presented in Table 2, the LOP of TRM samples was proven to be independent of the number of layers, while for the FRM composites, it can be concluded that crack flexural resistance decreased slightly with the addition of fiber. Thus, the FRM composite with 6% fiber had the highest LOP due to the lower w/c ratio and higher matrix volume (see Table 1). It must be remarked that the fibers in both types of composites slightly contributed to flexural resistance throughout the pre-cracking stage, given that the modulus of elasticity of the fibers was significantly lower (at least 10 times) than that of the matrix; however, the magnitude of the LOP was mainly governed by the strength of the matrix in each composite. Finally, it seems that the different distributions of the reinforcement—a homogenous fiber in FRM but a textile laminate in TRM—caused different stress distributions in the matrix, which, in turn, led to higher LOP values for FRMs. Furthermore, it should be mentioned that the higher amount of vegetable fibers in the TRM samples could increase matrix porosity, resulting in lower LOPs. Nonetheless, these partial conclusions regarding the magnitude of the LOP require more analysis and experimental evidence for confirmation.

On the other hand, the MOR values of TRM allow us to confirm that the addition of layers guaranteed a residual (post-cracking) flexural strength capacity, leading to a flexural hardening response of the composite (MOR_m_/LOP_m_ > 2.0, see Table 2). Furthermore, the results suggest that the optimal number of layers might range from 5 to 6. A drop in the bearing capacity of the 7-layer laminate could be due to insufficient impregnation of the increased number of layers as well as ineffective layers above the neutral axis. In fact, fiber agglomeration due to large amount of fabrics could weaken the interfacial transition zone within the matrix, making this area vulnerable to tension stresses, as reported in [67]. Furthermore, the use of 7-layer laminate with lower mortar/fabric thickness and higher (w/c)_final_ (see Table 1) resulted in an unbalanced relationship between the amount of matrix and fabric which, in turn, reduced the LOP and mechanical performance in the transition zone. It should be mentioned that laminates with 5–6 layers had the highest contribution to post-flexural resistance and highest MOR_m_/LOP_m_.

**Figure 6:**
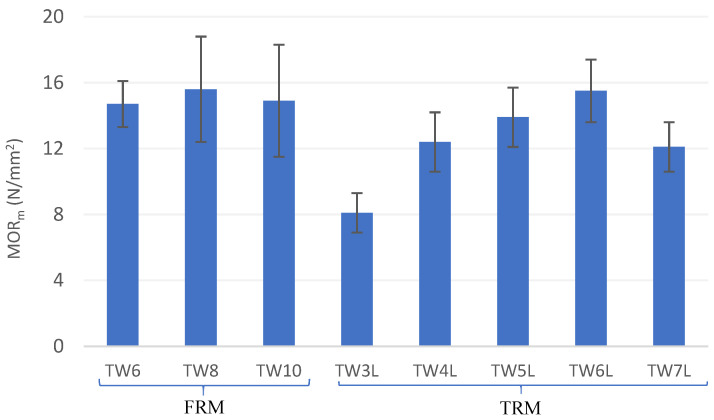
Results of Modulus of Rupture (MOR_m_) for the tested composites.

Likewise, the addition of short TW fibers provided a post-cracking flexural strength capacity (MOR_m_/LOP_m_ > 1.0) to the FRM composite. In this sense, fibers can bridge the cracks by controlling the opening and by ensuring a stress transfer mechanism across the crack height. The results highlight that the TW6 and TW10 composites presented similar MORs while the TW8 composites presented a 6% higher MOR at 28 days of curing (see Figure 6). Hence, MOR increased with an increase in the fiber content by up to 8% but decreased for greater fiber amounts due to technical difficulties associated with mixing, the balling effect, and compaction [5]. Similar results have been reported by Khorami et al. [68], in which the MOR of FRM reinforced by different amounts of waste kraft pulp fiber (1–14%) was investigated, for which it was shown that 8% fiber had the highest bending resistance. Nevertheless, although this material shows signs of post-cracking strength, its ductility and energy absorption capacity are limited and hence only nonstructural applications can be considered for this material.

**Figure 7:**
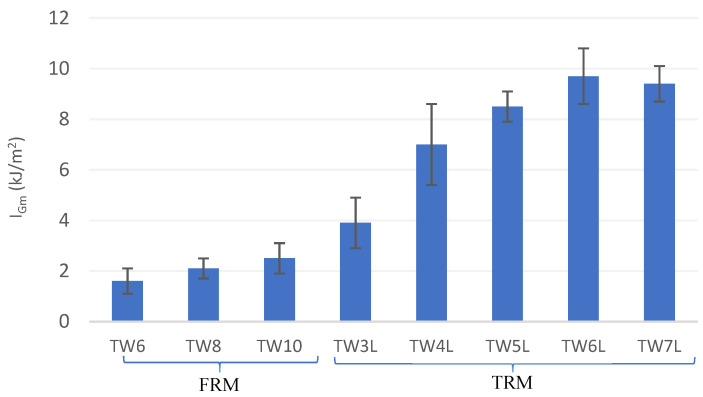
Results of toughness index (I_Gm_) for the tested composites.

Regarding toughness (I_Gm_), this parameter followed a similar tendency in TRM with respect to that obtained for MOR_m_, i.e., it was found that I_Gm_ reached maximum for an optimal number of 6 layers, with 148% higher values than TW3L. However, in FRM, the results presented an increase in I_Gm_ with fiber dosage, with TW10 having higher energy absorption (56% higher values than TW6). As shown in Figure 7, the toughness index of all TRM composites had a greater value than that of FRM due to the formation of multiple cracking; for instance, TW6L had more than four times greater energy absorption than TW8. Hence, the previous statement regarding the potential structural and nonstructural applicability of both materials is further reinforced by these results.

Finally, and only as a reference, the flexural stiffness of the pre-cracked zone (K_m_) was computed to quantify the deformability in the linear stages. The K parameter remained almost constant for FRM samples, decreasing by only 5% with the increase in fiber content, while in TRM composites, this parameter was rather independent of the number of layers and followed the same trend as LOP. Reinforcement in a nonwoven fabric form was proven to have a higher stiffness with respect to the short fibers, as K was more than twofold greater in TRM samples.

Overall, in the unaged samples, TW8 and TW6L had superior mechanical properties among FRMs and TRMs, respectively. Consequently, accelerated aging cycles were carried out on these composites to identify and quantify the damage and strength degradation of both FRM and TRM composites.

### 3.2. Flexural Test on Aged Composites

**Figure 8:**
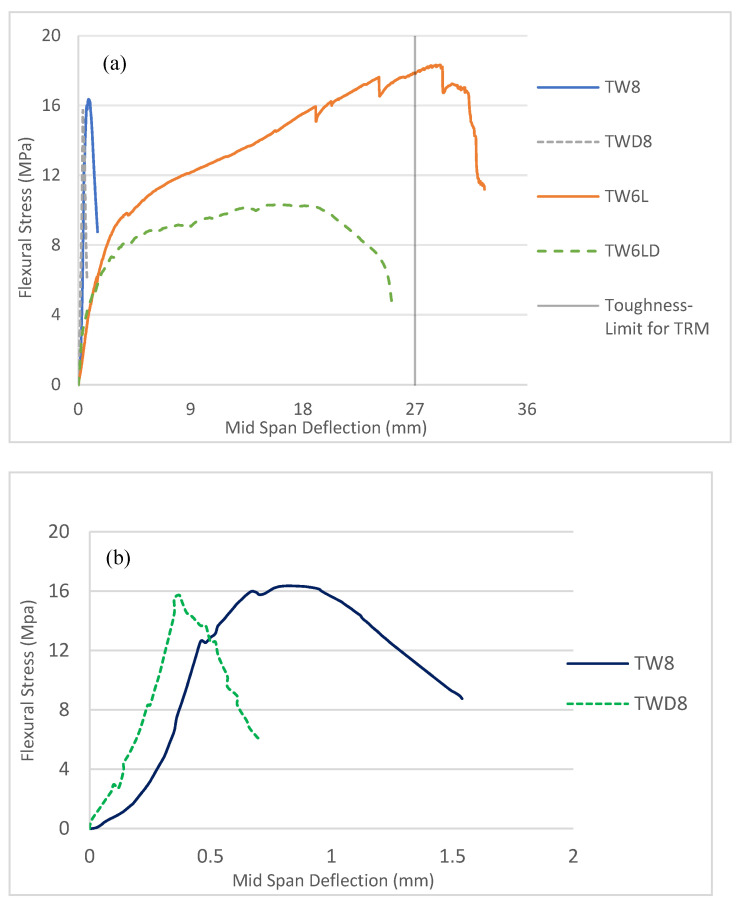
Representative flexural stress: deflection relationships obtained for aged samples for (**a**) all samples and (**b**) the FRM samples.

The results presented in Figure 8 and Table 3 allow us to confirm that the accelerated aging procedure negatively affected the post-cracking mechanical properties of the composites compared to those that were unaged. As expected, there was a loss of bending resistance in the composites after aging. In the textile laminate, the reduction of MOR* was about 35% (from 15.5 to 10 MPa), while this reduction was only 3% (from 15.6 to 15.2 MPa) for short reinforcement. Likewise, the reduction in the reinforcement contribution was 21 and 42% for FRM and TRM respectively, though the TW6LD still had a higher reinforcement contribution. It should be also mentioned that the TW short fibers were only made of cotton and polyester while TW fabric also contained flax. Thus, the amount of synthetic fiber—which is more durable than vegetable-based fibers—in the short reinforcement exceeded that of fabric fibers. When the fiber-reinforced composite was subjected to various wet-dry cycles the fibers, mainly vegetable fibers, lost adherence to the matrix due to reprecipitation of the hydrated compounds within the void space at the fiber-cement interface. Finally, full mineralization occurred, resulting in embrittlement of the vegetable fibers [65].

The reduction in toughness and energy absorption is considered one of the key matters of durability. This issue is related to an increase in fiber rupture and a decrease in fiber pull-out strength due to a combination of the weakening of the fibers by alkali attack, fiber mineralization, and volume variation due to the high water absorption of fibers. In this regard, the FRM and TRM samples experienced 42 and 30% I_Gm_* reductions, respectively. Nonetheless, due to the longer fiber length and more contact with the cement paste, the TRM sample could still develop a flexural hardening response with multiple cracking, though the slope of this zone was less than the corresponding unaged ones, demonstrating the loss in fiber stiffness due to degradation.

Finally, the elastic pre-cracking properties (LOP* and K*) presented on average unaltered or even higher values due to further cement hydration, as these are mainly dependent on the matrix, which is slightly affected by the aging procedure. Similar results have been reported by Claramunt et al. [69].

### 3.3. SEM Observations

**Figure 9:**
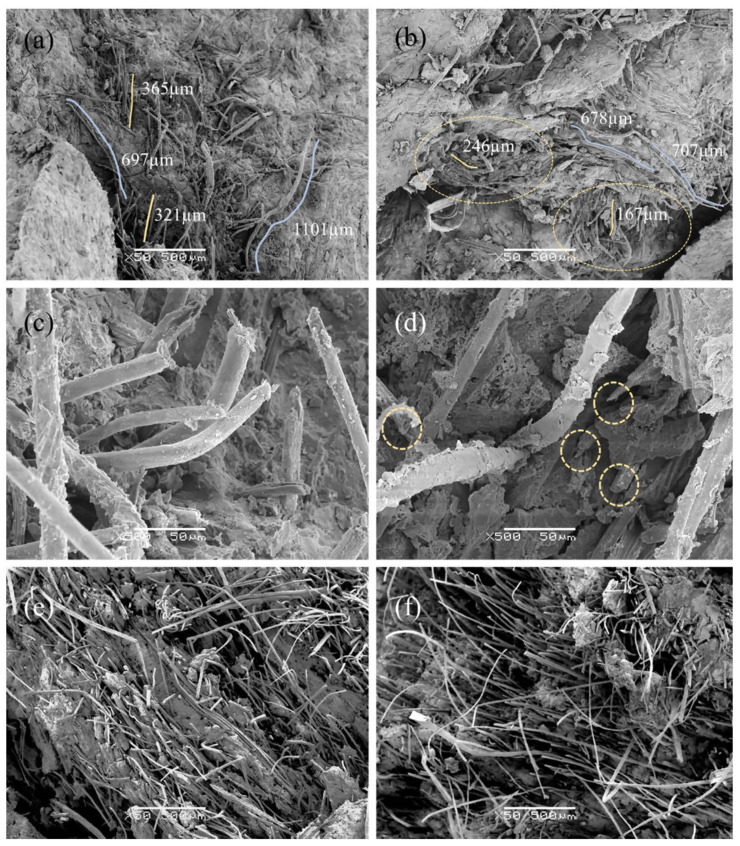
SEM micrographs of the fracture surfaces of the composites: (**a**) TW8; (**b**) TW8D; (**c**) TW8; (**d**) TW8D; (**e**) TW6L; and (**f**) TW6LD.

The loss of mechanical properties of the aged composites, mainly in absorbed energy and toughness, occurred due to the loss of adhesion and degradation of the vegetable fiber, as already explained. Both phenomena were more critical in the FRM than in the TRM due to the different distributions of the fibers. In the microimages of Figure 9 comparing the samples TW8 (Figure 9a) and TW8D (Figure 9b), the length of the fibers in the former are seen to be somewhat longer than those in the latter since most of the fibers in the aged samples were cut near the surface due to rupture. Therefore, the longer fibers generated more energy loss than the shorter ones through the pull-out mechanism of the fiber-cement interactions. Moreover, in Figure 9c,d, we could identify the differences between the fibers plucked from the unaged samples and the split fibers, indicated with a yellow circle, from the aged samples. Therefore, the wet-dry cycles induced damage, leading to an increase in the number of fibers failing due to rupture, thereby decreasing the fibers’ pull-out.

Nonetheless, regarding the TRM samples (see Figure 9e,f), the difference between the aged and unaged composites was insignificant since even after the accelerated aging, although properties were lost, a certain reinforcement effect was still maintained, which prevented breakage of the sample due to brittleness (see Figure 8a). Furthermore, longer fibers and the protrusion of a large set of fibers from the cement matrix were evident in TRM images with respect to FRM images. In fact, the fibers were dispersed randomly but homogeneously in the FRMs, while in the TRMs, the fibers were grouped in layers parallel to the surface, where a higher reinforcement density was obtained, which allowed for greater mechanical properties of this type of composite, mainly better energy absorption and toughness.

**Figure 10:**
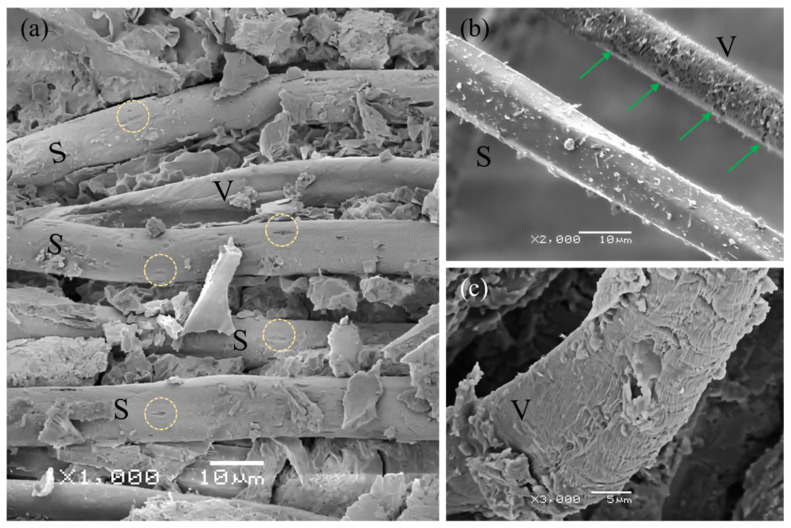
SEM micrographs of the fiber surfaces: (**a**) fibers from the broken section of an unaged sample; (**b**,**c**) fibers from the broken section of an aged sample.

In general, fibers in the unaged composites had clean surfaces whilst those in the aged composites appeared rougher and surrounded by precipitation products. In Figure 10a depicting the fibers from a broken section under unaged conditions, a set of synthetic fibers (S) can be observed that are clearly distinguished by their almost cylindrical shape, with some extrusion marks as indicated by a yellow circle. A vegetable fiber (V) is also distinguished by being more oblong due to its more irregular and hollow section. After the accelerated aging procedure, Figure 10b, we observed that the synthetic fiber (S) seemed to have greater durability since the surface does not appear to have been affected and only some cement hydration products appeared. By contrast, in the vegetable fiber (V), some damage with superficial cracks appeared, indicated by green arrows. Figure 10c shows the surface roughness caused by the accelerated aging treatment in detail.

### 3.4. Shrinkage

As can be seen in Table 4, the reference sample had the highest shrinkage strain. In general, hardened cement paste undergoes high drying shrinkage with respect to concrete or mortar as the changes in the volume of the latter are largely restrained by the rigidity of the aggregates [70]. In both types of reinforced composites, the incorporation of fibers led to a decrease in the amount of shrinkage. For instance, TW8 and TW6L could reduce the shrinkage of the paste by 44 and 30%, respectively. It seems that a higher percentage of vegetable fibers in textile reinforcement with respect to short random reinforcement (almost 10%) causes an increase in the matrix porosity, which leads to higher drying shrinkage due to higher water absorption.

## 4. Conclusions

The objective of this paper was to compare the mechanical performance of two types of cement composites reinforced by two forms of textile waste, either short random fibers or nonwoven fabrics. To identify potential applications of the resulting materials, the mechanical and durability properties of both FRM, composites with three different dosages of short fibers (6–10%), and TRM, laminates with 3–7 layers, were characterized. The following conclusions were derived from the results and may be limited to the scope of the current study:

- The pre-cracking flexural performances of both unaged 6-layer nonwoven TRM and FRM with 8% of fibers were comparable. However, the post-cracking flexural performance and especially the energy absorption capacity of the former were significantly superior (by four times) compared to the latter. The results suggest that randomly distributed short fibers from textile waste have limitations in terms of mechanical performance due to limited cracking capacity and maximum mixable amount of these fibers.
- Both composites were subjected to an accelerated aging process that primarily affected the energy absorption of the materials. Nonetheless, the toughness and stiffness of the aged TRM were greater (three times) than the aged FRM. SEM observations confirmed that accelerated aging was associated with an increase in fiber fracture and a decrease in fiber pull-out, especially in vegetable fibers, due to the alkali attack. Nonetheless, modifying the matrix with pozzolanic materials such as silica fume could improve the durability of this composite.

Finally, the results for the 6-layer TRM panel as the most prominent TW composite—showing a flexural strength of 15.5 MPa and a toughness of 9.7 kJ/m^2^—demonstrate that application of this type of waste is technically feasible and could be potentially used for reinforcement of nonstructural constructs (e.g., facade panels, roofing, raised floors, and masonry structures). The application of 6-layer TRM panels as a façade cladding is currently under investigation due to its potential benefits in terms of sustainability (cost and environmental and social impacts).

We highlight that these results and conclusions are preliminary and incipient, since the experimental program is in its early stages. An increase in the statistical population of experimental results is expected to shed more light on and reinforce some of the preliminary conclusions stated herein.

## Figures and Tables

**Table 1:** Physical properties of the composites (FRM and TRM: Fiber- and Textile-Reinforced Mortar).

Composite Type	Code	(w/c)_final_	Cement (g)	Fiber (g)	Fiber Weight Fraction (%)	Thickness (mm)	Mortar/Fabric Thickness	No. of Specimens
FRM	TW6	0.40	1600	96	6	20	-	6
	TW8	0.50	1400	112	8	20	-	6
	TW10	0.50	1200	120	10	20	-	6
	TW8D	0.45	1400	112	8	20	-	6
TRM	TW3L	0.40	1350	42	3.1	6.5	1.95	6
	TW4L	0.40	1500	56	3.7	8.5	1.83	6
	TW5L	0.40	1530	70	4.9	9.2	1.48	6
	TW6L	0.40	1550	84	5.4	10.0	1.22	6
	TW7L	0.45	1600	98	6.1	10.2	0.92	6
	TW6LD	0.40	1474	84	5.7	10.0	1.22	12

**Table 2:** Results of all of the Textile Waste composites at 28 days (Coefficient of Varuation in %).

Composite Type	Code	LOP_m_ (N/mm^2^)	MOR_m_ (N/mm^2^)	I_Gm_ (kJ/m^2^)	K_m_ (GPa)	MOR_m_/LOP_m_
TRM	TW3L	4.1 (29)	8.1 (14)	3.9 (28)	8.7 (17)	2.0
	TW4L	4.5 (24)	12.4 (14)	7.0 (24)	10.7 (14)	2.8
	TW5L	4.1 (24)	13.9 (13)	8.5 (7)	7.8 (26)	3.4
	TW6L	4.6 (19)	15.5 (12)	9.7 (12)	11.3 (21)	3.4
	TW7L	4.2 (12)	12.1 (12)	9.4 (8)	10.6 (21)	2.8
FRM	TW6	12.7 (12)	14.7 (9)	1.6 (31)	4.0 (19)	1.1
	TW8	11.1 (10)	15.6 (20)	2.1 (19)	3.9 (15)	1.4
	TW10	10.7 (10)	14.9 (23)	2.5 (24)	3.7 (19)	1.4

**Table 3:** Test results on the aged composites (CoV in %)(* indicates the mechanical parameters after the accelerated aging).

Samples	LOPm* (N/mm^2^)	MORm* (N/mm^2^)	I_Gm_* (kJ/m^2^)	K_m_* (GPa)	MORm*/LOPm*
TW8D (FRM)	13.3 (11)	15.2 (10)	1.2 (21)	4.0 (17)	1.10
TW6LD (TRM)	5.1 (31)	10.0 (27)	6.8 (33)	12.0 (5)	1.96

**Table 4:** Results for shrinkage strain of tested samples.

Samples	Reference	TW6	TW8	TW10	TW3L	TW6L
Max Shrinkage (microstrain)	2560	1490	1420	1370	2550	1870
Time (days)	160	56	56	56	84	84

## Data Availability

The data presented in this study are available upon request from the corresponding author.
